# A Distinct Pattern of Circulating Amino Acids Characterizes Older Persons with Physical Frailty and Sarcopenia: Results from the BIOSPHERE Study

**DOI:** 10.3390/nu10111691

**Published:** 2018-11-06

**Authors:** Riccardo Calvani, Anna Picca, Federico Marini, Alessandra Biancolillo, Jacopo Gervasoni, Silvia Persichilli, Aniello Primiano, Hélio José Coelho-Junior, Maurizio Bossola, Andrea Urbani, Francesco Landi, Roberto Bernabei, Emanuele Marzetti

**Affiliations:** 1 Fondazione Policlinico Universitario Agostino Gemelli, IRCCS, Rome 00168, Italy; riccardo.calvani@gmail.com (R.C.); jacopo.gervasoni@policlinicogemelli.it (J.G.); silvia.persichilli@policlinicogemelli.it (S.P.); mauriziobossola@gmail.com (M.B.); andrea.urbani@unicatt.it (A.U.); francesco.landi@unicatt.it (F.L.); roberto.bernabei@unicatt.it (R.B.); emanuele.marzetti@policlinicogemelli.it (E.M.); 2 Università Cattolica del Sacro Cuore, Rome 00168, Italy; anielloprim@gmail.com (A.P.); coelhojunior@hotmail.com.br (H.J.C.-J.); 3 Department of Chemistry, Sapienza University of Rome, Rome 00168, Italy; federico.marini@uniroma1.it (F.M.); alessandra.biancolillo@uniroma1.it (A.B.); 4 Applied Kinesiology Laboratory–LCA, School of Physical Education, University of Campinas, Campinas-SP 13.083-851, Brazil

**Keywords:** aging, muscle, protein, metabolism, metabolomics, profiling, biomarkers, multi-marker, physical performance, multivariate

## Abstract

Physical frailty and sarcopenia (PF&S) are hallmarks of aging that share a common pathogenic background. Perturbations in protein/amino acid metabolism may play a role in the development of PF&S. In this initial report, 68 community-dwellers aged 70 years and older, 38 with PF&S and 30 non-sarcopenic, non-frail controls (nonPF&S), were enrolled as part as the “BIOmarkers associated with Sarcopenia and Physical frailty in EldeRly pErsons” (BIOSPHERE) study. A panel of 37 serum amino acids and derivatives was assayed by UPLC-MS. Partial Least Squares–Discriminant Analysis (PLS-DA) was used to characterize the amino acid profile of PF&S. The optimal complexity of the PLS-DA model was found to be three latent variables. The proportion of correct classification was 76.6 ± 3.9% (75.1 ± 4.6% for enrollees with PF&S; 78.5 ± 6.0% for nonPF&S). Older adults with PF&S were characterized by higher levels of asparagine, aspartic acid, citrulline, ethanolamine, glutamic acid, sarcosine, and taurine. The profile of nonPF&S participants was defined by higher concentrations of α-aminobutyric acid and methionine. Distinct profiles of circulating amino acids and derivatives characterize older people with PF&S. The dissection of these patterns may provide novel insights into the role played by protein/amino acid perturbations in the disabling cascade and possible new targets for interventions.

## 1. Introduction

Over the last decades, sarcopenia, the progressive and generalized decline in skeletal muscle mass and function with age, has become a “blockbuster” condition in geriatrics, given its increasing prevalence in a globally aging world and its clinical relevance [1,2,3,4]. Indeed, this condition conveys a broad spectrum of negative health-related outcomes, including disability, loss of independence, institutionalization, and mortality [5,6]. Frailty has been defined as a geriatric “multidimensional syndrome characterized by decreased reserve and diminished resistance to stressors,” and is often envisioned as a pre-disability condition [7]. Sarcopenia overlaps with the clinical picture of frailty, especially in its physical domain, and may represent both the biological substratum of physical frailty (PF) and the pathophysiologic basis upon which the negative health outcomes of PF develop [8,9]. The two conditions have therefore been merged into a new entity (i.e., PF and sarcopenia; PF&S) [10] that was operationalized in the context of the “Sarcopenia and Physical fRailty IN older people: multi-componenT Treatment strategies” (SPRINTT) project [11,12]. 

Although the pathophysiology of PF&S is complex and multifactorial, the central role attributed to muscle wasting suggests that biomarkers related to sarcopenia may be used to support the diagnosis and track the evolution of PF&S, unveil its underlying mechanisms, and identify meaningful targets for interventions [13,14].

Dietary protein intake and circulating amino acids play a pivotal role in muscle plasticity and trophism [15], but also modulate several biological processes (including inflammation, insulin sensitivity, and redox homeostasis) that may be involved in age-related muscle atrophy and dysfunction [16,17]. Hence, perturbations in protein-amino acid metabolism may represent a major mechanism in sarcopenia [18,19].

Amino acid profiling, especially when coupled with multivariate statistical analysis, may serve as a powerful analytical approach to explore the possible role of protein-amino acid networks in PF&S [20]. Recently, distinct amino acid signatures were associated with muscle mass in older adults with functional limitations [21] and low muscle quality [22] in the Baltimore Longitudinal Study of Aging. Moreover, reduced non-fasting plasma concentrations of the branched-chain amino acids (BCAAs) leucine and isoleucine were detected in Norwegian older community-dwellers with sarcopenia [23], while higher proline concentrations were independently associated with sarcopenia in older Japanese people [24]. Finally, low plasma levels of essential amino acids (EAAs) characterized the amino acid profile of severely frail Japanese older people compared with non-frail peers [25].

The “BIOmarkers associated with Sarcopenia and Physical frailty in EldeRly pErsons” (BIOSPHERE) study was designed to determine and validate a panel of PF&S biomarkers encompassing systemic inflammation, oxidative stress, muscle remodeling, neuromuscular junction dysfunction, and amino acid metabolism through multivariate statistical modeling [26]. In the present work, we report the initial results obtained through the simultaneous analysis of an array of circulating amino acids and derivatives coupled with Partial Least Squares–Discriminant Analysis (PLS-DA). This innovative approach allowed identifying distinct patterns of circulating amino acids and derivatives that characterize older adults with and without PF&S. This may represent a first relevant step towards the integration of specific biochemical measurements into the assessment of PF&S in research and clinical settings.

## 2. Materials and Methods

### 2.1. Study Design and Population

BIOSPHERE was conceived as a cross-sectional, case-control study [26]. The study protocol was approved by the Ethics Committee of the Università Cattolica del Sacro Cuore (Rome, Italy; protocol number: 8498/15) and is thoroughly described elsewhere [26]. Briefly, after obtaining written informed consent, 200 older persons, 100 cases (individuals with PF&S) and 100 non-physically frail, non-sarcopenic (nonPF&S) controls aged 70+ were enrolled. Selection criteria are reported in Table S1. Candidates were diagnosed with PF&S when presenting the following parameters: (a) low physical performance, defined as a summary score on the Short Physical Performance Battery (SPPB) [27] between 3 and 9; (b) low appendicular muscle mass (aLM) according to the criteria recommended by the Foundation for the National Institutes of Health (FNIH) sarcopenia project [28]; and (c) absence of major mobility disability, operationalized as an inability to walk 400 m in 15 min at a usual pace [29]. This initial analysis involved 68 participants (38 cases and 30 controls) in whom circulating amino acids and derivatives were measured.

### 2.2. Measurement of Appendicular Lean Mass by Dual X-Ray Absorptiometry (DXA)

Whole-body DXA scans were obtained on a Hologic Discovery A densitometer (Hologic, Inc., Bedford, MA, USA). Scan acquisition and analysis were performed according to manufacturer’s directions. Candidates were considered to be eligible if presenting with an aLM to body mass index (BMI) ratio (aLM_BMI_) <0.789 or <0.512 in men and women, respectively. When the aLM_BMI_ criterion was not met, candidates were tested with the alternative criterion (i.e., crude aLM < 19.75 kg in men and <15.02 kg in women) [28].

### 2.3. Blood Sample Collection

Blood samples were collected in the morning by venipuncture of the median cubital vein after overnight fasting, using commercial collection tubes (BD Vacutainer^®^; Becton, Dickinson and Co., Franklin Lakes, NJ, USA). For serum separation, samples were left at room temperature for 20 min and subsequently centrifuged at 1000× *g* for 10 min at 4 °C. Aliquots of serum were subsequently stored at −80 °C until analysis.

### 2.4. Amino Acids Profiling

Thirty-seven amino acids and derivatives (1-methylhistidine, 3-methylhistidine, 4-hydroxyproline, α-aminobutyric acid, β-alanine, β-aminobutyric acid, γ-aminobutyric acid, alanine, aminoadipic acid, anserine, arginine, asparagine, aspartic acid, carnosine, citrulline, cystathionine, cystine, ethanolamine, glutamic acid, glycine, histidine, isoleucine, leucine, lysine, methionine, ornithine, phenylalanine, phosphoethanolamine, phosphoserine, proline, sarcosine, serine, taurine, threonine, tryptophan, tyrosine, valine) were measured in serum through a ultraperformance liquid chromatography/mass spectrometry (UPLC/MS) validated methodology. Briefly, 50 µL of sample were mixed with 100 µL 10% (*w*/*v*) sulfosalicylic acid containing an internal standard mix (50 µM) (Cambridge Isotope Laboratories, Inc., Tewksbury, MA, USA) and centrifuged at 1000× *g* for 15 min. Ten microliters of the supernatant were transferred into a vial containing 70 µL of borate buffer to which 20 µL of AccQ Tag reagents (Waters Corporation, Milford, MA, USA) were subsequently added. Samples were then vortexed for 10 s and heated at 55 °C for 10 min. The chromatographic separation was performed by ACQUITY H-Class (Waters Corporation) using a CORTECS UPLC C18 column 1.6 µm 2.1 × 150 mm (Waters Corporation) eluted at a flow rate of 500 µL/min with a linear gradient (9 min) from 99 to 1 water 0.1% formic acid in acetonitrile 0.1% formic acid. The mass spectrometer was an ACQUITY QDa single quadrupole equipped with electrospray source operating in positive mode (Waters Corporation). The analytical process was monitored using amino acid controls (level 1 and level 2) manufactured by the MCA laboratory of the Queen Beatrix Hospital (Winterswijk, The Netherlands).

### 2.5. Statistical Analysis

All analyses were performed using in-house routines running under MATLAB R2015b environment (The MathWorks, Natick, MA, USA).

#### 2.5.1. Descriptive Statistics

Differences in demographic, anthropometric, clinical, and functional characteristics between cases and controls were assessed via *t*-test statistics and χ^2^ or Fisher exact tests, for continuous and categorical variables, respectively. All tests were two-sided, with statistical significance set at *p* < 0.05. 

#### 2.5.2. Partial Least Squares–Discriminant Analysis

The strategy for the identification and validation of potential biomarkers for PF&S relied on the building of discriminant models to differentiate cases from controls. The approach chosen for the present study was based on PLS-DA [30], because of its versatility and ability to deal with highly correlated predictors. Briefly, PLS-DA is a classification method based on the PLS regression algorithm [31]. PLS-DA builds the linear relation between a set of responses Y and a matrix of predictors X by projecting the latter onto a low-dimensional space of latent (abstract) variables (LVs) that are characterized by having the highest covariance with the responses to be predicted. The statistical reliability of the PLS-DA model was subsequently verified by a double cross-validation (DCV) procedure and by means of randomization tests [32]. Three figures of merit were considered in the present study: (i) the number of misclassifications (NMC); (ii) the area under the receiver operating characteristic (ROC) curve (AUROC); and (iii) the value of the discriminant Q2 (DQ2) [33].

For the identification of potential biomarkers, two approaches aimed at highlighting the experimental variables contributing the most to the classification model were followed, and they involved inspecting variable importance in projection (VIP) indices [31] and rank product (RP) [34], respectively. A more detailed description of the PLS-DA statistics is provided as supplementary material.

## 3. Results

### 3.1. Descriptive Characteristics of the Study Population

The study population included 38 older adults with PF&S and 30 nonPF&S controls. The main demographic, anthropometric, clinical, and functional characteristics of the study population according to the presence of PF&S are presented in Table 1. No differences between groups were observed with regard to age, gender distribution, number of co-morbid conditions, and number of prescription medications. The distribution of specific disease conditions and the prevalence of use of individual drug classes are shown in Table S2. As expected, physical performance, as assessed by the SPPB, was lower in PF&S participants (SPPB score: 7.4 ± 1.5) relative to controls (11.3 ± 0.9) (*p* < 0.0001). Similarly, aLM, either absolute or adjusted for BMI, was smaller in the PF&S group compared with nonPF&S enrollees.

### 3.2. Participant Classification According to PLS-DA

**Figure 1:**
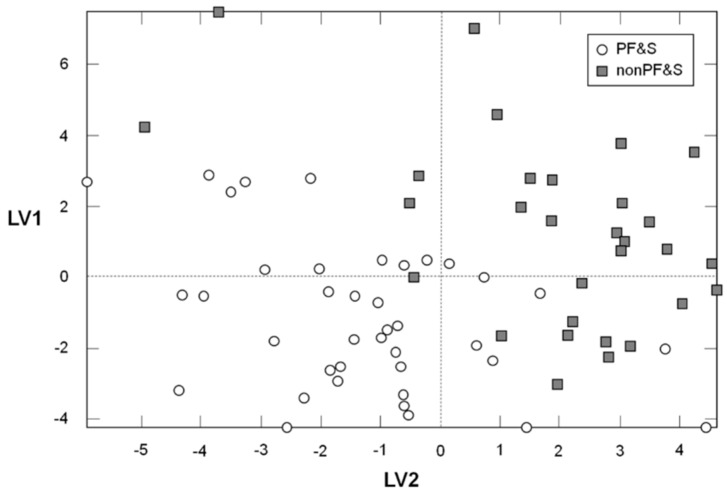
Scores plot showing the separation of participants according to the serum concentrations of amino acids and derivatives in the space spanned by the two latent variables (LV1 and LV2), as determined by Partial Least Squares–Discriminant Analysis (PLS-DA).

In order to verify the existence of a specific pattern of amino acids in participants with PF&S, a PLS-DA classification model was constructed and validated. The optimal PLS-DA model was built using three LVs that accounted for more than 44% of the variance originally present in the X block. As indicated by the DCV procedure, the model allowed to correctly predict the presence of PF&S in 95.7 ± 2.1% of participants in the calibration phase (94.7 ± 3.8% for PF&S and 96.7 ± 4.6% for controls), 84.1 ± 2.7% in the internal validation stage (82.6 ± 3.6% for PF&S and 86.0 ± 4.8% for controls), and 76.6 ± 3.9% in external validation (75.1 ± 4.6% for PF&S; 78.5 ± 6.0% for nonPF&S). Figure 1, which depicts the projection of participants onto the space spanned by the first two LVs of the PLS-DA model, shows a clear separation between participants with and without PF&S. 

**Figure 2:**
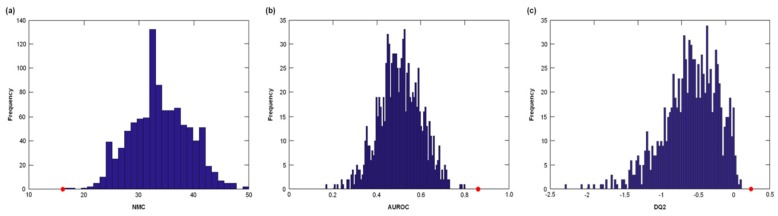
Distribution of (**a**) number of misclassifications (NMC), (**b**) area under the receiver operating characteristic (ROC) curve (AUROC), and (**c**) discriminant Q2 (DQ2) values under their respective null hypothesis as estimated by permutation tests (blue histograms) and the corresponding values obtained by the PLS-DA model on unpermuted data (red circles). Values obtained on the real dataset (red circles) fall outside of the corresponding null hypothesis distribution (blue histograms), corresponding to a *p* < 0.05.

The classification ability of the PLS-DA model was further validated by comparing the results of the DCV with the distributions of NMC, AUROC and DQ2 under the null hypothesis (Figure 2). For each of the three figures of merits considered, the values obtained on the real dataset fell outside of the corresponding null hypothesis distribution, which corresponds to a *p* < 0.05.

In order to identify the metabolites that were mostly involved in discriminating between cases and controls, the values of the VIP indices were inspected. The variables corresponding to a VIP greater than one are reported in Table 2. Nine amino acids were found to contribute significantly to the discrimination model. Participants with PF&S were characterized by higher levels of asparagine, aspartic acid, citrulline, ethanolamine, glutamic acid, sarcosine, and taurine. Conversely, the profile of non-PF&S individuals was defined by higher levels of α-aminobutyric acid (AABA) and methionine. Serum concentrations of non-discriminant amino acids are reported in Table S3.

## 4. Discussion

In the present study, we report the first results from the BIOSPHERE study. The most relevant finding was that older individuals with PF&S showed a distinct profile of circulating amino acids characterized by higher serum levels of asparagine, aspartic acid, citrulline, ethanolamine, glutamic acid, sarcosine, and taurine. Conversely, the profile of nonPF&S participants was defined by higher levels of AABA and methionine.

The existence of an amino acid signature in the setting of PF&S suggests that specific metabolic alterations might be involved in the pathogenesis of this condition. Indeed, PF&S was associated with lower circulating levels of the EAA methionine. EAAs are defined as those amino acids that must be provided with the diet to meet optimal requirements [35]. The reduction of serum concentrations of a number of EAAs (including methionine) with age was reported in both genders and was purportedly associated with decreases in total energy and protein intake [36]. In addition, low plasma levels of EAA were found in severely frail older people [25]. These findings may be linked to malnutrition (both quantitative and qualitative), a common causative factor of frailty and sarcopenia [37,38]. The concomitant low serum concentration of the non-essential non-proteinogenic amino acid AABA seems to corroborate the previous finding since AABA may derive from the catabolism of methionine [39]. Furthermore, plasma levels of AABA were found to be associated with both the quality and amount of dietary protein [40,41]. Although these findings seem to point towards a poor-quality protein diet or (selective) malabsorption, further studies are needed to clarify the relationship between diet and circulating EAA levels in the context of PF&S.

Methionine is also involved in one-carbon metabolism, a crucial pathway that modulates multiple physiologic processes, including nucleotide biosynthesis, amino acid homeostasis, epigenetic maintenance, and redox balance [42]. Not surprisingly, alterations in one-carbon metabolism were observed in aging and age-related diseases, such as cancer, cardiovascular disease, and neurodegeneration [42,43]. Sarcosine, the *N*-methyl-derivative of glycine, is another relevant intermediate of one-carbon metabolism [42]. Sarcosine is formed from dietary choline and the metabolism of methionine [44,45], and can be found in muscles and other body tissues. A recent metabolomics study showed that circulating sarcosine levels were reduced with aging both in rodents and humans, while dietary restriction prevented this decline in both species [46]. Counterintuitively, sarcosine levels were higher in persons with PF&S relative to controls. However, circulating sarcosine may increase in case of folate deficiency, because folate mediates the conversion of sarcosine to glycine [45]. Thus, this finding might be linked to insufficient folate ingestion and/or perturbation in folate/one-carbon metabolism. 

Sarcosine also activates autophagy in mouse fibroblasts in a dose-dependent manner [46], and alterations in myocyte quality control mechanisms (including autophagy) may contribute to sarcopenia [47,48,49]. In particular, defective autophagic clearance of damaged cellular constituents, alterations in mitochondrial proteostasis and dynamics, and impaired mitochondriogenesis are thought to be critically involved in age-related muscle degeneration [50]. In this context, the presence of ethanolamine among the most discriminant metabolites for PF&S classification is of particular interest. Ethanolamine is a naturally occurring amino alcohol that plays a pivotal role in the synthesis of phosphatidylethanolamine, a central intermediate of lipid metabolism and a major component of biological membranes [51]. Phosphatidylethanolamine is also directly involved in the regulation of autophagy [52], and it is postulated that ethanolamine treatment or the consumption of ethanolamine-rich foods may increase cellular phosphatidylethanolamine levels, induce autophagy, and provide beneficial anti-aging effects across species [52]. While serum ethanolamine levels were different between PF&S and controls, this did not result in a corresponding difference in serum phosphatidylethanolamine concentrations, suggesting alterations in CDP-ethanolamine pathway, the major route of phosphatidylethanolamine production [53]. Interestingly, the disruption of CDP-ethanolamine pathway in muscle was associated with alterations in mitochondrial biogenesis and muscle atrophy in mice [54].

Taurine is a ubiquitous non-proteinogenic sulfur-containing amino acid that represents the most abundant free amino acid in the heart, retina, skeletal muscle, brain, and leukocytes, accounting for approximately 0.1% of total body weight [55]. In skeletal muscle, which contains 70% of total body taurine, this amino acid is involved in the regulation of ion channel function, membrane stability, mitochondrial quality control, and calcium homeostasis [56,57,58,59]. In muscle, taurine also serves osmoregulatory, anti-oxidant, and anti-inflammatory functions [56,57,58,59]. Given these multiple actions, taurine has recently been proposed as a candidate therapeutic agent against sarcopenia [60]. While it is reported that serum taurine concentrations decline with age in men [36], increased levels of serum taurine have been retrieved in the metabolic profiles of old wild-type mice from different genetic backgrounds [61]. Circulating levels of taurine are regulated by the balance among different factors, including dietary intake, intestinal absorption, bile acid conjugation, urinary excretion, and endogenous synthesis from methionine and cysteine [55]. Taurine may be released from cells following osmotic perturbations, oxidative stress, and (chronic) inflammatory stimulation [58]. Further studies are needed to unveil the mechanisms responsible for the high circulating taurine levels observed in older adults with PF&S.

Citrulline is a non-essential non-protein amino acid with a key role in nitrogen homeostasis [62]. Citrulline is an end product of glutamine metabolism and an endogenous precursor of arginine [63]. For its capacity of promoting endothelial nitric oxide availability and vasodilation, “sparing” arginine and glutamine from hepatic catabolism and the supposed ability to activate mTORC1 signaling [64], citrulline was proposed as a pharmaconutrient to counteract sarcopenia [65]. Several reports have shown that serum citrulline increases with age [36,66,67]. In addition, in a metabolomics study assessing the individual variability in human blood metabolites [68], citrulline was among the circulating molecules that exhibit a remarkable age-related increase. The authors attributed this finding to impairment in urea cycle efficiency due to the progressive decline of liver and renal function with age [68]. However, no differences in kidney or liver function were observed between participants belonging to the two BIOSPHERE study groups. Further investigation on interorgan nitrogen homeostasis pathways are needed to explain the higher circulating values of citrulline found in older adults with PF&S. 

Asparagine, aspartic acid, and glutamic acid are among the six amino acids that are metabolized in resting muscles [69]. These amino acids provide the amino groups and the ammonia required for the synthesis of glutamine and alanine, which are released following protein meals and in the post-absorptive state [69]. The carbon skeletons of these metabolites may be used solely for de novo synthesis of TCA-cycle intermediates and glutamine [70]. The higher levels of asparagine, aspartic acid, and glutamic acid observed in persons with PF&Ss may be suggestive of perturbations in muscle energy metabolism associated with muscle wasting. Interestingly, a pattern of metabolic changes accompany muscle remodeling after disuse, including energy substrate accumulation (e.g., asparagine) in atrophied muscles [71,72]. 

As opposed to EAAs, no significant differences were observed between groups in the serum levels of BCAAs. It should however be considered that the absorption of dietary proteins is influenced by several factors, which may impact their bioavailability and circulating concentrations. In particular, whether PF&S is associated with changes in the expression of amino acid transporters and gastrointestinal physicochemical properties is presently unknown. Furthermore, the lower splanchnic extraction of BCAAs might offset subtle differences in their systemic concentrations between groups [73]. Notwithstanding, our finding on BCAAs is not consistent with previous investigations that reported changes in BCAA concentrations in relation to sarcopenia, low muscle mass, and functional limitation [21,22,23]. These discrepancies may be due to differences in operational definitions adopted and experimental designs among studies. In addition, heterogeneity in eating habits among participants of the different studies may contribute to the contrasting results.

The present study has some limitations that should be acknowledged. First, the study population was relatively small, and a great number of experimental variables were included in the analyses. However, the innovative analytical approach implemented in the study, based on PLS-DA plus DCV, is an ideal strategy to cope with this issue. The study sample was exclusively comprised of Caucasian individuals, which impedes generalizing the findings to other ethnic groups. Other factors that might affect circulating amino acid levels include lifestyle and eating habits [74,75]. For instance, regular participation in physical activity has been associated with reduced circulating levels of BCAAs as well as alanine and proline across a wide age spectrum [76]. Furthermore, exercise training has shown to increase the plasma levels of glycine and citrulline in overweight adults [77]. Although only people not engaged in regular exercise were enrolled in the present study, the amount of physical activity of participants was not quantified. Hence, the possible influence of physical activity on amino acid profiles in the context of PF&S could not be established. The same applies to the possible influence of different nutritional patterns and amino acid intakes. However, as recently highlighted, differences in circulating amino acids are less marked than those between amino acid intakes [74]. The cross-sectional design of the study does not allow inference to be drawn on the time course of changes of the variables considered and on cause-effect relationships. Finally, although a fairly large number of amino acids and derivatives was assayed, it cannot be excluded that more powerful biomarkers of PF&S might be obtained through the analysis of a larger range of biomediators.

## 5. Conclusions

In the present study, a PLS-DA-based approach allowed distinct patterns of circulating amino acids and derivatives to be identified in older persons with and without PF&S. The pathways unveiled by this initial investigation may be used to generate new mechanistic hypotheses on the pathophysiology of PF&S. Furthermore, the longitudinal implementation of the proposed analytical strategy could facilitate the tracking of PF&S condition over time and the monitoring of response to treatments. This may represent a first relevant step towards the integration of specific biochemical measurements into the assessment of PF&S, both in clinical and research settings.

## Figures and Tables

**Table 1:** Main characteristics of BIOmarkers associated with Sarcopenia and Physical frailty in EldeRly pErsons (BIOSPHERE) participants according to the presence of physical frailty and sarcopenia (PF&S).

	PF&S (*n* = 38)	nonPF&S (*n* = 30)	*p*
Age, years (mean ± SD)	76.4 ± 4.9	74.6 ± 4.3	0.1067
Gender (female), *n* (%)	25 (65.8)	16 (53.3)	0.4280
BMI, kg/m^2^ (mean ± SD)	29.1 ± 4.4	26.7 ± 2.4	0.0112
SPPB (mean ± SD)	7.4 ± 1.5	11.3 ± 0.9	<0.0001
aLM, kg (mean ± SD)	16.2 ± 3.2	19.4 ± 3.9	0.0004
aLM_BMI_ (mean ± SD)	0.554 ± 0.120	0.795 ± 0.264	<0.0001
Number of disease conditions * (mean ± SD)	2.3 ± 1.5	1.8 ± 1.4	0.1448
Number of medications (mean ± SD)	3.2 ± 1.8	2.8 ± 1.9	0.4115

* Includes hypertension, coronary artery disease, prior stroke, peripheral vascular disease, diabetes, chronic obstructive pulmonary disease, and osteoarthritis. BMI: body mass index; SPPB: Short Physical Performance Battery; aLM: appendicular lean mass; PF&S: physical frailty and sarcopenia; nonPF&S: non physically frail, non sarcopenic; SD: standard deviation.

**Table 2:** Serum concentrations of discriminant analytes, variable importance in projection (VIP) values, and rank product (RP) values in BIOSPHERE participants with and without physical frailty and sarcopenia (PF&S). Serum concentrations are shown as mean ± standard deviation.

	PF&S (*n* = 38)	nonPF&S (*n* = 30)	VIP	RP
α-aminobutyric acid (µmol/L)	20.0 ± 4.9	22.3 ± 5.7	2.2	8.0
Asparagine (µmol/L)	91.0 ± 12.6	77.8 ± 13.4	3.4	2.0
Aspartic Acid (µmol/L)	24.6 ± 5.4	17.0 ± 4.0	5.8	2.6
Citrulline (µmol/L)	44.8 ± 12.1	36.8 ± 11.5	2.1	2.8
Ethanolamine (µmol/L)	10.3 ± 1.7	9.0 ± 2.2	1.7	9.9
Glutamic acid (µmol/L)	71.7 ± 16.6	54.3 ± 21.2	2.3	8.5
Methionine (µmol/L)	22.6 ± 2.8	23.4 ± 5.7	1.3	6.3
Sarcosine (µmol/L)	1.9 ± 0.6	1.5 ± 0.5	1.4	8.0
Taurine (µmol/L)	220.1 ± 36.5	189.5 ± 47.2	1.8	6.7

## Supplementary Materials

The following are available online at http://www.mdpi.com/2072-6643/10/11/1691/s1, Partial Least Squares–Discriminant Analysis; Table S1: Eligibility criteria in BIOSPHERE; Table S2: Distribution of co-morbid conditions and prevalence of use of individual drug classes in BIOSPHERE participants with and without physical frailty & sarcopenia (PF&S); Table S3: Serum concentrations of non-discriminant analytes in BIOSPHERE participants with and without of physical frailty & sarcopenia (PF&S).
