# Supplementary material for: A Distinct Pattern of Circulating Amino Acids Characterizes Older Persons with Physical Frailty and Sarcopenia: Results from the BIOSPHERE Study
Source: Nutrients. 2018 Nov 6;10(11):1691. doi: 10.3390/nu10111691 (PMC6265849; doi:10.3390/nu10111691)
Supplement: Supplementary file 1 [file nutrients-10-01691-s001.zip › nutrients-363658-supp2.docx]

Supplementary Materials: A Distinct Pattern of Circulating Amino Acids Characterizes Older Persons with Physical Frailty and Sarcopenia: Results from the BIOSPHERE Study

**Table S1.** Eligibility criteria in BIOSPHERE.

| **PF&S** | **nonPF&S** |
| --- | --- |
| **Inclusion criteria** | |
| Men and women aged ≥70 years | Men and women aged ≥70 years |
| SPPB score between 3 (included) and 9 (included) | SPPB score >9 |
| Sedentary lifestyle | Sedentary lifestyle |
| Low muscle mass according to the cut-points indicated by the FNIH sarcopenia project * | Normal muscle mass according to the FNIH sarcopenia project |
| Ability to complete the 400-metre walk test within 15 minutes without sitting, use of any assistive device or help of another person | Ability to complete the 400-metre walk test within 15 minutes without sitting, use of any assistive device or help of another person |
| **Permanent exclusion criteria for both cases and controls** | |
| Inability or unwillingness to provide informed consent | |
| Nursing home residence | |
| Current diagnosis of schizophrenia, other psychotic or bipolar disorder | |
| Consumption of more than 14 alcoholic drinks per week | |
| Self-reported inability to walk across a room | |
| Difficulty communicating with the study personnel due to speech, language, or hearing problems | |
| MMSE <24 | |
| Severe arthritis (e.g., awaiting joint replacement) that would interfere with the ability to perform physical performance testing | |
| Cancer requiring treatment in the past three years, except for non-melanoma skin cancers or cancers that have an excellent prognosis (e.g., early stage breast or prostate cancer) | |
| Lung disease requiring regular use of corticosteroids or supplemental oxygen | |
| Severe cardiovascular disease (including NYHA class III or IV congestive heart failure, clinically significant valvular disease, history of cardiac arrest, presence of an implantable defibrillator, or uncontrolled angina) | |
| Parkinson’s disease or other progressive neurological disorder | |
| Renal disease requiring dialysis | |
| Chest pain, severe shortness of breath, or occurrence of any other safety concerns during the 400-metre walk test | |
| Other medical, psychiatric, or behavioural factors that in the investigator's judgment may interfere with study participation | |
| Other illnesses of such severity that life expectancy is less than 12 months | |
| **Temporary exclusion criteria for both cases and controls** ** | |
| Uncontrolled hypertension (systolic blood pressure >200 mmHg, or diastolic blood pressure >110 mmHg) | |
| Uncontrolled diabetes with recent weight loss, diabetic coma, or frequent hypoglycaemia | |
| Stroke, hip fracture, hip or knee replacement, or spinal surgery in the past six months | |
| Serious conduction disorder (e.g., third-degree heart block) | |
| Uncontrolled arrhythmias, new Q waves within the past six months or ST-segment depression (>3mm) on the ECG | |
| Myocardial infarction, major heart surgery (i.e., valve replacement or bypass surgery) in prior six months | |
| Deep vein thrombosis or pulmonary embolism in the past six months | |

* Low muscle mass was defined as a crude appendicular lean mass (ALM) <19.75 kg in men and <15.02 kg in women or as an ALM-to-body mass index ratio <0.789 in men and <0.512 in women.

** Participants who were excluded for one or more of the temporary medical conditions listed above could be rescreened after a period considered clinically appropriate by the study physician.

*Abbreviations*: FNIH, Foundation for the National Institutes of Health; MMSE, Mini Mental State Examination; NYHA, New York Heart Association; nonPF&S. non physically frail, non sarcopenic; PF&S, physical frailty and sarcopenia; SPPB, short physical performance battery.

**Table S2.** Distribution of co-morbid conditions and prevalence of use of individual drug classes in BIOSPHERE participants with and without of physical frailty & sarcopenia (PF&S).

|  | **PF&S (*n* = 38)** | **nonPF&S (*n* = 30)** | ***p*** |
| --- | --- | --- | --- |
| Co-morbid conditions, *n* (%) |  |  |  |
| Cardiovascular disease * | 27 (71) | 20 (66.6) | 0.6975 |
| Cerebrovascular disease | 3 (7.9) | 2 (6.6) | 0.8474 |
| Chronic lung disease | 7 (18.4) | 5 (16.6) | 0.8495 |
| Musculoskeletal conditions | 28 (73.6) | 21 (70) | 0.7367 |
| Diabetes mellitus | 8 (21) | 6 (20) | 0.9164 |
| Cancer | 6 (15.7) | 4 (13.3) | 0.7759 |
| Drugs, *n* (%) |  |  |  |
| Analgesics | 9 (23.7) | 6 (20) | 0.7163 |
| Antiaggregants | 10 (26.3) | 6 (20) | 0.5419 |
| Anticoagulants | 8 (21.0) | 6 (20) | 0.9164 |
| Antidepressants | 5 (13.2) | 3 (10.0) | 0.6882 |
| Antidiabetics | 10 (26.3) | 8 (26.6) | 0.9747 |
| Antihypertensives | 22 (57.9) | 16 (53.3) | 0.7072 |
| Antiosteoporotic agents | 3 (7.9) | 1 (3.3) | 0.4273 |
| Antiulcers | 8 (21.0) | 5 (16.7) | 0.6475 |
| Benzodiazepines | 8 (21.0) | 6 (20) | 0.9164 |
| Bronchodilators | 7 (18.4) | 5 (16.7) | 0.8495 |
| Diuretics | 8 (21.0) | 8 (26.6) | 0.5876 |
| Laxatives | 13 (34.2) | 10 (33.3) | 0.9382 |
| Lipid-lowering agents | 2 (5.3) | 1 (3.3) | 0.7004 |
| Nonsteroidal anti-inflammatory drugs | 8 (21.0) | 4 (13.3) | 0.4071 |

***** Includes high blood pressure, coronary artery disease, congestive heart failure, atrial fibrillation

**Table S3.** Serum concentrations of non-discriminant analytes in BIOSPHERE participants with and without of physical frailty & sarcopenia (PF&S).

|  | **PF&S (*n* = 38)** | **nonPF&S (*n* = 30)** |
| --- | --- | --- |
| β-alanine (µmol/L) | 7.0 ± 1.9 | 6.5 ± 2.6 |
| β-amino butyric acid (µmol/L) | 1.9 ± 1.3 | 1.7 ± 1.1 |
| γ-aminobutyric acid (µmol/L) | 0.4 ± 0.1 | 0.3 ± 0.1 |
| 1-methylhistidine (µmol/L) | 7.4 ± 9.9 | 9.2 ± 16.7 |
| 3-methylhistidine (µmol/L) | 5.1 ± 1.8 | 5.2 ± 2.5 |
| 4-hydroxyproline (µmol/L) | 20.2 ± 10.1 | 17.2 ± 7.9 |
| Alanine (µmol/L) | 393.3 ± 73.1 | 384.3 ± 98.3 |
| Aminoadipic acid (µmol/L) | 1.4 ± 0.7 | 1.4 ± 0.8 |
| Arginine (µmol/L) | 114.4 ± 22.2 | 103.7 ± 31.2 |
| Cystine (µmol/L) | 33.1 ± 14.0 | 32.4 ± 11.3 |
| Glycine (µmol/L) | 284.0 ± 75.7 | 260.9 ± 65.6 |
| Histidine (µmol/L) | 78.9 ± 9.8 | 82.2 ± 11.4 |
| Isoleucine (µmol/L) | 60.1 ± 13.7 | 63.2 ± 18.3 |
| Leucine (µmol/L) | 122.4 ± 19.2 | 121.9 ± 27.0 |
| Lysine (µmol/L) | 200.0 ± 27.6 | 196.8 ± 41.6 |
| Ornithine (µmol/L) | 120.3 ± 26.1 | 109.4 ± 25.0 |
| Phenylalanine (µmol/L) | 71.0 ± 9.0 | 66.1 ± 11.0 |
| Phosphoethanolamine (µmol/L) | 1.3 ± 1.0 | 1.6 ± 0.8 |
| Proline (µmol/L) | 202.5 ± 56.0 | 199.9 ± 44.4 |
| Serine (µmol/L) | 128.0 ± 19.5 | 118.7 ± 16.9 |
| Threonine (µmol/L) | 127.4 ± 26.5 | 125.1 ± 22.3 |
| Tryptophan (µmol/L) | 60.7 ± 9.0 | 62.0 ± 13.1 |
| Tyrosine (µmol/L) | 68.3 ± 12.5 | 65.7 ± 14.6 |
| Valine (µmol/L) | 226.4 ± 38.3 | 221.2 ± 42.9 |
