# Supplementary material for: A Distinct Pattern of Circulating Amino Acids Characterizes Older Persons with Physical Frailty and Sarcopenia: Results from the BIOSPHERE Study
Source: Nutrients. 2018 Nov 6;10(11):1691. doi: 10.3390/nu10111691 (PMC6265849; doi:10.3390/nu10111691)
Supplement: Supplementary file 1 [file nutrients-10-01691-s001.zip › nutrients-363658-supp1.docx]

Supplementary Materials: A Distinct Pattern of Circulating Amino Acids Characterizes Older Persons with Physical Frailty and Sarcopenia: Results from the BIOSPHERE Study

**Partial Least Squares–Discriminant Analysis**

Partial Least Squares–Discriminant Analysis (PLS–DA) is a classification method based on the PLS regression algorithm [1]. In order for the method to also be used in classification where the responses to be predicted are of a qualitative and not quantitative nature (in the present study, whether the participant is a case or a control), y is coded as a ‘dummy’ binary vector, assuming the value 1 if the participant has physical frailty and sarcopenia (PF&S) and 0 if he/she is a control. The classification of the individuals is then operated on the basis of the predicted value of y, adopting a threshold value of 0.5. If the predicted y is above that value, the individual is classified as belonging to PF&S group, while if it is below, he/she is predicted to be a control.

To identify and validate potential biomarkers for PF&S, a classification approach relying on the possibility of building discriminant models was followed. Among the possible discriminant classification techniques, PLS–DA [2] was chosen due to its characteristics of allowing to obtain reliable models also in cases where there can be many (and possibly correlated) predictors. This advantage stems directly from PLS–DA being based on the PLS regression algorithm [1]. In PLS, the linear calibration model between the responses **Y** and the predictors **X** is defined in a low-dimensional subspace of latent (abstract) variables (LVs). In order to be as effective as possible for the calibration problem, these LVs are characterized by being the directions along which there is the highest covariance between the responses and the predictors. The use of the PLS algorithm to cope also with discriminant classification issues, where the responses to be predicted are of qualitative and not quantitative in nature (in the present study, whether the participant is a case or a control), requires the resposes to be suitably coded to account for class belonging. This is accomplished by introducing a ‘dummy’ binary vector **y**, which carries the class information about the individuals: for each participant, the corresponding response will assume the value 1 or 0 depending on whether he/she has PF&S or is a control. A PLS regression model is then calculated between the experimental matrix **X** and the dummy coded **y**, and the discrimination of the samples is carried out on the basis of the predicted response. However, it should be stressed that, while the “true” response is binary coded, the predicted value of y is real-valued and so a criterion should be defined in order to classify the individuals. In the present study, a threshold value of 0.5 was adopted, so that if the predicted y was above that value, the individual was classified as belonging to PF&S group, while if it was below, he/she was predicted to be a control.

To safeguard against the risk of overoptimistic evaluation of the classification performances and of identification of chance biomarkers, the statistical reliability of the PLS-DA model was subsequently verified by a double cross-validation (DCV) procedure and by means of randomization tests [3]. Double cross-validation is a variant of standard cross-validation which makes use of two (an outer and an inner) resampling loops instead of a single one. The outer loop is meant to mimic an external (not based on individuals employed for parameter selection) test set to be used for the validation of a PLS-DA model, the optimal complexity of which is chosen on the basis of the minimum classification error on the inner loop samples. Furthermore, since, especially when not many individuals are analyzed, good classification results could be obtained also in cases where there should be no relevant difference between the classes, due to sampling, the statistical significance of the results of PLS-DA modeling was assessed by comparing the values of selected figures of merit with their respective non-parametric distributions under the null hypothesis, estimated by means of randomization tests. In particular, three figures of merit were considered in the present study: (i) the number of misclassifications (NMC); (ii) the area under the receiver operating characteristic (ROC) curve (AUROC); and (iii) the value of the discriminant Q2 (DQ2) [4]. NMC corresponds to the total number of classification errors (i.e., PF&S participants predicted as controls and vice versa). AUROC is a measure of the discriminatory power of a binary classifier and can assume values between 1 (perfect classification) and 0 (no discrimination). DQ2 is a modification of the standard Q2 that was introduced to cope with the peculiarities of classification problems addressed by regression methods [5]. Like its regression analogue, DQ2 assumes its highest values (close to 1) in the case of a perfect discrimination between classes, but it is not bound to the 0–1 range of values (i.e., it can also be negative).

For the identification of potential biomarkers, two approaches aimed at highlighting the experimental variables contributing the most to the classification model were followed, and they involved inspecting the so–called variable importance in projection (VIP) indices [1] and rank product (RP) [6], respectively. VIP scores indicate the contribution of each of the predictors to the variance in the response accounted for by the PLS-DA model and are scaled so that a “greater than 1” rule can be used to assess statistical significance. On the other hand, RP is an index accounting for how consistently a variable emerges as relevant in a resampling procedure and, as the name suggests, involves a criterion for model-based ranking of the predictors. The absolute value of the PLS-DA regression coefficient is used to define the discriminant ability of the predictors, which are then ranked: at each cross-validation split, metabolites are ranked in decreasing order of discriminant ability, so that the variable with the highest discriminant power is given rank 1, the next largest 2 and so on. Eventually, RP is defined for each metabolite as the geometric mean of its ranks in all cross-validation segments; variables with the lowest rank products are those considered to be discriminant.

**References**

1. Wold, S.; Martens, H.; Wold, H. The multivariate calibration problem in chemistry solved by the PLS method. In *Matrix Pencils. Lecture Notes in Mathematics*, vol 973; Publisher: Springer, Berlin, Germany, 1983; pp. 286–293, ISBN: 978-3-540-11983-8.

2. Barker, M.; Rayens, W. Partial least squares for discrimination. *J. Chemom.* **2003**, *17*, 166–73, doi:10.1002/cem.785.

3. Westerhuis, J. A.; Hoefsloot, H. C. J.; Smit, S.; Vis, D. J.; Smilde, A. K.; van Velzen, E. J. J.; van Duijnhoven, J. P. M.; van Dorsten, F. A. Assessment of PLSDA cross validation. *Metabolomics* **2008**, *4*, 81–9, doi:10.1007/s11306-007-0099-6.

4. Szymańska, E.; Saccenti, E.; Smilde, A. K.; Westerhuis, J. A. Double-check: validation of diagnostic statistics for PLS-DA models in metabolomics studies. *Metabolomics* **2012**, *8*, 3–16, doi:10.1007/s11306-011-0330-3.

5. Westerhuis, J. A.; van Velzen, E. J. J.; Hoefsloot, H. C. J.; Smilde, A. K. Discriminant Q2 (DQ2) for improved discrimination in PLSDA models. *Metabolomics* **2008**, *4*, 293–6, doi:10.1007/s11306-008-0126-2.

6. Smit, S.; van Breemen, M. J.; Hoefsloot, H. C. J.; Smilde, A. K.; Aerts, J. M. F. G.; de Koster, C. G. Assessing the statistical validity of proteomics based biomarkers. *Anal. Chim. Acta* **2007**, *592*, 210–7, doi:10.1016/j.aca.2007.04.043.
